# Molecular Perspectives for mu/delta Opioid Receptor Heteromers as Distinct, Functional Receptors 

**DOI:** 10.3390/cells3010152

**Published:** 2014-03-05

**Authors:** Edmund W. Ong, Catherine M. Cahill

**Affiliations:** 1 Department of Biomedical and Molecular Sciences, Queen’s University, Kingston, Ontario K7L 3N6, Canada; E-Mail: edmund.ong@queensu.ca; 2 Department of Anesthesiology and Perioperative Care, University of California Irvine, 2125 Gillespie Neuroscience, 837 Health Science Road, Irvine, CA 92697, USA

**Keywords:** Mu opioid receptor, Delta opioid receptor, G protein coupled receptor, oligomerization, heteromer, trafficking, internalization, receptor signaling

## Abstract

Opioid receptors are the sites of action for morphine and the other opioid drugs. Abundant evidence now demonstrates that different opioid receptor types can physically associate to form heteromers. Understandings of the nature, behavior, and role of these opioid receptor heteromers are developing. Owing to their constituent monomers’ involvement in analgesia, mu/delta opioid receptor (M/DOR) heteromers have been a particular focus of attention. There is now considerable evidence demonstrating M/DOR to be an extant and physiologically relevant receptor species. Participating in the cellular environment as a distinct receptor type, M/DOR availability is complexly regulated and M/DOR exhibits unique pharmacology from that of other opioid receptors (ORs), including its constituents. M/DOR appears to have a range of actions that vary in a ligand- (or ligands-) dependent manner. These actions can meaningfully affect the clinical effects of opioid drugs: strategies targeting M/DOR may be therapeutically useful. This review presents and discusses developments in these understandings with a focus on the molecular nature and activity of M/DOR in the context of therapeutic potentials.

## 1. Introduction and Historical Perspective

Opioid drugs are widely used and have remained the first line of therapy for moderate to severe pain for well over a century. Opioid actions are mediated via the opioid receptors (ORs), a family of three G-protein coupled receptors (GPCRs): mu opioid receptor (MOR), delta opioid receptor (DOR), and kappa opioid receptor (KOR). ORs (and GPCRs in general) are large integral membrane polypeptides with complex tertiary structures featuring seven transmembrane domains and both intra- and extracellular loops and termini. ORs are functional at the cell surface, where they associate with complex intracellular signaling machinery primarily via coupling to heterotrimeric G-proteins. Opioid agonists bind to and activate ORs, which in turn activate G-proteins, thereby, propagating intracellular signaling cascades, which ultimately inhibit cell responsiveness. Primarily, this involves inhibition of adenylyl cyclase (and so, cAMP), inhibition of N- and P/Q-type voltage-gated calcium channels, and activation of potassium channels (thereby, depolarizing the cell), with additional effects on kinases and transcription factors.

ORs and their ligands were originally classified by pharmacological type (MOR, DOR, KOR) and subtype, of which general consensus held there to be eight: MOR1,2,3; DOR1,2; KOR1,2,3. This pharmacological understanding failed to harmonize with the identification, in the early-to-mid 1990s, of only three OR cDNAs—one each for MOR, DOR, and KOR. Interestingly, multiple splice variants of MOR have since been reported, including truncated forms (for review, see [1]). Some of these splice variants may have implications for clinical opioid effectiveness [2]. Nevertheless, these variants have pharmacologies either equivalent to prototypical MOR or markedly different (e.g., MOR-1K has been reported to be excitatory and active intracellularly, but not surface-available). As such, their existence fails to account for OR subtype pharmacology. 

Pharmacological investigations of the ORs also revealed an array of synergisms and interactions between the ORs (for review, see [3]). Such ‘spooky’ action at a sibling and the discrepancy between the pharmacologically and genetically identifiable OR species led to interest in whether ORs, like several other GPCRs, undergo heteromerization. In this process, multiple different receptors associate to form complexes (heteromers; for reviews, see [4,5,6]). In some cases, such as the GABA B receptor, such complexing is required for functionality [7,8], while in others it serves to alter functionality, such as the interactions of dopamine D_2_ and adenosine A_2A_ receptors [9].

OR heteromerization was first described by Jordan and Devi [10], who showed KOR and DOR to be capable of interacting to form functional K/DOR heteromers. Following that first report, the existence of OR heteromers has been demonstrated by a large, and still growing, body of literature utilizing a range of experimental techniques including immunoblotting and co-immunoprecipitation, immunocytochemistry, and bioluminescence and Foerster resonance energy transfer [11,12,13,14,15,16,17]. These proximity and interaction assay findings are convincing and have been thoroughly reviewed [3,18]. OR heteromers are also a leading explanation for the generation of multiple pharmacological subtypes from only three mRNAs.

Of the species identified, the M/DOR heteromer has emerged as the subject of greatest interest. This is in large part because most clinically used opioid drugs act on MOR, and preclinical results have demonstrated both MOR and DOR to have analgesic effects. The heteromer generated by their interaction holds promise as a therapeutic target and, as discussed below, appears to be involved in changes to the opioid system following chronic MOR agonist exposure (e.g., chronic opioid therapy). In this review, we discuss the present understanding of M/DOR and show how these findings are most appropriately synthesized by considering M/DOR as a distinct functional unit with unique regulation and activity.

### Nomenclature

A definitive consensus regarding the appropriate nomenclature for receptor heteromers and their disambiguation from non-heteromer receptor-receptor interactions has yet to emerge. In this review, we use the abbreviation form M/DOR, K/DOR, *etc.* when referring to the receptor heteromers and the form MOR-DOR, KOR-DOR, *etc.* when referring to non-heteromer receptor-receptor interaction.

## 2. Formation

### 2.1. Molecular Nature of Mu-Delta Interaction

Understanding the details of an interaction’s quaternary structure is typically challenging and notably so in the case of OR heteromers. The constituent monomers are relatively large membrane-bound proteins with individually complex tertiary structures; they undergo many conformational changes (which are themselves poorly understood) as part of their normal function; and they are individually fully competent, meaning that their interacting state may occur but is not required. A definitive identification of structural nature of the interaction would typically require a method such as crystallography of the complete heteromer. That result remains elusive due to the complexities involved.

Indeed, the crystallographic structures of the OR monomers have only recently been reported ([19,20,21,22] for commentary, see [23,24]). These findings will soon permit the refinement of the currently most useful techniques for understanding the molecular nature of the OR heteromers: computational modeling and experimental disruption.

#### 2.1.1. Computational

Most conceptualizations of OR heteromers are as contact dimers. That is, two adjacent independent polypeptides abutting each other. Their overall interaction is a product of several specific interactions between residues of each monomer and results in conformational changes to each. Current computational studies have sought to identify both the specific interacting residues and broadly how the secondary and tertiary structures of each receptor interact.

Even considering only broad domain-domain interactions, there are a large number of potential interacting configurations for heteromers of receptors each with seven transmembrane domains. Filizola and colleagues [25] predicted probable configurations by first identifying correlated mutations; residues on each MOR and DOR, which tend to undergo apparently coordinated mutations due to a need to maintain interactions. The resulting set of probabilistically identified residues was then sorted for residues that would be sterically available for interactions. Using models of the receptors’ transmembrane domains based on the crystallographic structure of rhodopsin, the authors retained only those residues exposed on the outer surface of those domains. The locations of this final set of interacting residues were then used to identify the probable domain-domain interactions in M/DOR. Nearly all of the interacting residues were in TM1 of MOR and TM4, TM5, and TM6 of DOR.

A later study by Liu and colleagues [26] constructed 3D models of the complete sequences MOR and DOR. This computationally-intensive work addressed a major limitation of Filizola *et al.*’s earlier study, which was limited to considering only the transmembrane domains. However, even these full-length models were still not based upon OR crystallographic structures, which had not yet been produced. They were, instead, homology models; each section of the models was based on the known structures of other proteins with similar sections. The resulting models were then subjected to several simulated interactions in a variety of configurations to find the most likely (sterically and energetically favorable) interacting structure. The authors concluded that the most favorable interaction was between TM1 of MOR and TM4 of DOR and also involved both carboxyl terminal domains. These findings are in agreement with those of experiments which disrupted MOR-DOR associations via alterations to either TM1 of MOR or the carboxyl tails of either OR (see below).

It is notable that both of these computational studies arrived at similar conclusions, identifying the same regions of MOR and DOR as interacting, while using distinct methodologies. However, both studies were undertaken prior to the generation of OR crystallographic structures and are limited by their extrapolation to MOR and DOR of data of the structures of other proteins. In particular, both analyses are heavily reliant on the crystallographic structure of rhodopsin, which limits the importance of their consilience. Much more convincing validations of these analyses, their models, and their findings are now possible using the recently identified crystallographic structures of the OR monomers. Data and modeling derived from these structures are expected to greatly improve understandings of OR heteromerization (also see [24]).

#### 2.1.2. Experimental Disruption

Several studies have provided insight into the nature of the heteromeric interactions as a result of specific interventions which disrupted the heteromer. In regards to interactions between transmembrane domains, He *et al.* [27] constructed a mutant MOR with a duplicate TM3 in place of TM1. When coexpressed with DOR in HEK293 cells, non-mutated MOR formed M/DOR heteromers identifiable by coimmunoprecipitation, while the mutant MOR did not interact with DOR. Conversely, the authors created a construct, which expressed only TM1 of MOR with a GFP tag. When coexpressed with DOR in HEK293 cells, this TM1-GFP construct formed an interaction with DOR, which was detectable by coimmunoprecipitation. This suggested that TM1 of MOR was both required for MOR-DOR heteromerization and sufficient, independently, to physically interact with DOR. Interestingly, the authors extended upon the latter finding by generating a MOR(TM1)-TAT fusion protein which was delivered intracellularly following *in vivo* administration and could be used to competitively disrupt M/DOR heteromers (discussed below).

Separately, the carboxyl-terminal domains of both MOR and DOR appear to play a role in determining the pharmacology of the M/DOR heteromer. Fan *et al.* [15] and others reported that M/DOR exhibits pharmacology (ligand binding and activation) unique from that of either constituent alone. In particular, these authors noted that coexpression of MOR and DOR altered the binding profiles of DAMGO (a MOR agonist) and DPDPE (a DOR agonist) as compared to expression of MOR or DOR alone, respectively. The authors then constructed truncated mutants of MOR and DOR, removing carboxyl-terminal amino acids (23 or 35 in MOR; 15 or 28 in DOR). When truncated DOR was coexpressed with MOR, DAMGO binding was normalized. Similarly, when truncated MOR was coexpressed with DOR, DPDPE binding was normalized. Notably, while c-terminal truncation altered heteromer pharmacology, it had little impact on coimmunoprecipitation. This is consistent with MOR-DOR interactions also involving the transmembrane domains. 

A subsequent study from this group [28] used multiple mutant variants of MOR and DOR to identify specific amino acids involved in the MOR-DOR interaction. By moving from mutants with longer truncations to versions with single amino acid changes, the authors identified a three residue sequence (GGG) near the carboxyl terminus of DOR which was required for MOR-DOR interaction. No such required sequence was found for the MOR c-terminal tail. Additionally, this study also identified a three residue sequence (SVR) on the third intracellular loop of both MOR and DOR, which was also required for MOR-DOR interaction. This region has previously been shown to be involved with MOR coupling to g-proteins. These findings, which differ slightly from those of Fan’s earlier study, may be attributable to the method used to detect MOR-DOR interactions, which relied upon cotrafficking of MOR and DOR in response to a nuclear localization sequence inserted into one of the pair. Different methodologies used in the literature to test heteromerization probe different related aspects of the interaction, including here: physical association (e.g., coimmunoprecipitation and involving TM1 of MOR), pharmacology (e.g., ligand binding, g-protein coupling, and involving the c-terminal tails of MOR and DOR), and cotrafficking (involving specific carboxyl-terminal residues of DOR and possibly g-protein interactions). 

#### Summary, Molecular Nature of Mu-Delta Interaction

Taken together, the present studies investigating the molecular nature of the MOR-DOR interaction suggest that a set of several residues in several domains of each MOR and DOR interact. Interacting residues appear to be present in transmembrane domains, intracellular loops, and carboxyl tails. Further, overlapping subsets of these residues/domains are involved with different aspects of the interaction. That is, it is constructive to view the MOR-DOR interaction as having multiple facets rather than as a binary (independent or interacting) state.

It is anticipated that the recently identified crystallographic structures of the OR monomers will lead to the refinement of and improved confidence in understandings of OR heteromerization.

### 2.2. Coexpression of MOR and DOR

MOR-DOR interactions resulting in the formation of M/DOR heteromers obviously require coexpression of MOR and DOR. Such coexpression has, for some time, been reported to be common and widespread [29]. However, a recent study reported substantially less coexpression of MOR and DOR. Using a transgenic mouse expressing a knock-in DOR linked to enhanced green fluorescent protein (eGFP), Scherrer and colleagues [30] immunolabeled eGFP and MOR. They reported that colocalization of DOR-linked eGFP and MOR signals occurred in less than five percent of DRG neurons labeled for either OR. However, subsequent studies addressing this inconsistency have continued to report extensive coexpression of MOR and DOR. Wang and colleagues [31] used single-cell PCR, *in situ* hybridization, and immunofluorescence to demonstrate coexpression of MOR and DOR in DRG neurons. Further, these authors also demonstrated single-cell electrophysiological responses to both MOR- and DOR-selective ligands. This latter finding is mirrored by work by Chieng and Christie [32] who used electrophysiological recordings from neurons of the central nucleus of the amygdala to show widespread single-cell responsiveness to both MOR- and DOR-selective ligands. Similarly, Beaudry and colleagues [33] showed that both MOR- and DOR-selective agonists acted on primary afferents to inhibit formalin- and capsaicin-evoked substance P release. Interestingly, subsequent development of this transgenic construct has produced an animal expressing both DOR-eGFP, as well as MOR linked to red fluorescent protein. Preliminary descriptions of work using this double fluorescent knock-in have reported meaningful coexpression of MOR and DOR in a number of brain structures [34].

A compelling line of reasoning to explain these inconsistent results was presented by Stockton and Devi [18] and focuses on three separate findings. First, that the labeling observed with DOR-eGFP is substantially different from that observed with DOR-myc when both are immunolabeled via their linked tag. The eGFP-linked DOR is found primarily on the cell surface, while the myc-tagged DOR is distributed at intracellular sites [35]. Second, that the DOR-eGFP mice are known to overexpress DOR-eGFP in comparison to wild-types [36]. Finally, third, that increased cell surface DOR leads to a decrease in MOR maturation ([17], discussed below). In this argument, DOR-eGFP expression and surface localization are excessive and result in suppressed MOR expression. This would result in a bias among cells coexpressing MOR and DOR towards being detected as expressing only DOR. Such a bias would be further compounded by the high detection limit of the fluorescence microscopy methodology used; imaging parameters appropriate for capturing the intense, amplified signals from high-MOR-expressing cells could fail to distinguish low-to-moderate-MOR-expressing cells (potentially including the suppressed-MOR-expressing coexpressing cells) from background. This would ultimately result in, and account for, the underreporting of MOR-DOR coexpression.

#### Summary, Coexpression of MOR and DOR

The extent of neuronal coexpression of MOR and DOR remains the subject of some contention as a result of apparently contradictory experimental findings. The degree to which MOR and DOR are coexpressed and the circumstances which influence their coexpression must obviously affect the physiological role of M/DOR. Current findings regarding both the abundance and lack of MOR-DOR coexpression have methodological limitations, which have slowed the emergence of a consensus regarding the extent of coexpression.

### 2.3. Induction of M/DOR Formation

Though widely described in heterologous systems, the relevance of endogenous OR heteromers was, for some time, difficult to establish. Using newly-generated antibodies against the M/DOR heteromer, we and our co-authors demonstrated the existence of endogenous M/DOR heteromers [37]. These monoclonal antibodies against M/DOR were generated using a subtractive immunization strategy and screened for reactivity to M/DOR, as well as MOR, DOR, and the expression system used. This process produced a monoclonal antibody, which specifically recognized an epitope of M/DOR. Using this antibody, we and our co-authors identified endogenous M/DOR in neurons from primary dorsal root ganglia (DRG) cultures, as well as in cells of the medial nucleus of the trapezoid body (MNTB) and the rostral ventral medulla (RVM). These structures are involved in peripheral sensory transmission, audition, and pain perception, respectively. M/DOR were not detected in either MOR- or DOR-knock-out animals.

Further, we and our co-authors showed endogenous M/DOR abundance to vary in response to extracellular conditions, indicating MOR-DOR interactions to be regulated at the cellular level. Chronic, but not acute, morphine treatment of cultured DRG neurons resulted in increased MOR-DOR colocalization and increased M/DOR abundance, as detected both immunofluorescently and by ELISA. Similarly, chronic *in vivo* morphine administration resulted in increased M/DOR abundance in the cortex, MNTB, RVM, hypothalamus, nucleus accumbens, and ventral tegmental area. Similar results were found following chronic treatment with naltriben, a DOR antagonist. These finding demonstrated both the *in vivo* presence of M/DOR and the regulation of M/DOR in response to exogenous ligands.

Interestingly, we, and others, have previously demonstrated that chronic morphine treatment causes a translocation of DOR from intracellular sites to the plasma membrane of treated neurons [32,38,39]. This process requires MOR presence [40] and does not alter DOR mRNA levels [41]. It now appears that the changes in DOR distribution described following chronic morphine are, in fact, part of an upregulation of M/DOR formation. Notably, the stability of DOR mRNA levels suggests this regulation to be post-translational. That is, existing MOR and DOR are induced to interact.

#### Summary, Induction of M/DOR formation

M/DOR have been directly detected in neuronal structures involved in sensory transmission, audition, and pain perception. M/DOR abundance varies in response to extracellular conditions; specifically, chronic, but not acute, morphine treatment increases M/DOR abundance. The regulation of M/DOR formation may involve control over the interaction of existing ORs, rather than *de novo* synthesis.

### 2.4. Subcellular Location of Formation

The subcellular location, or locations, at which M/DOR formation takes place has been difficult to discern. Law and colleagues [42] attempted to directly address the question using a heterologous system to co-express wild-type MOR or DOR with surface-transport deficient mutants of DOR or MOR, respectively. That is, one of the MOR-DOR pair was a mutant incapable of being trafficked to the cell surface. The authors then used fluorescence-activated cell sorting (FACS) to identify surface expression of each OR. This study found that neither wild-type OR was able to rescue its surface trafficking deficient partner. The authors therefore conclude that M/DOR formation occurs only at the cell surface. Alternatively, these results may also indicate that the formation of a M/DOR heteromer from a surface trafficking deficient mutant results in a surface trafficking deficient heteromer. 

#### Summary, Subcellular Location of Formation

The location of heteromer formation remains unclear.

## 3. Outwards Trafficking

### 3.1. Protein Chaperone

As evidenced by the responsiveness of M/DOR abundance to cellular conditions without altering individual OR transcription/translation, M/DOR formation is a controlled process. Further, given that increased M/DOR abundance following chronic morphine exposure is accompanied by translocation of DOR from internal to cell-surface sites, it is likely that M/DOR formation and this DOR outwards trafficking are linked processes. While Law and colleagues [42] suggest that heteromer formation occurs only at the cell surface, they did not address the possibility that M/DOR formation may occur internally but resultant heteromers are retained intracellularly. Indeed, this is the case for DOR; over 60% of newly synthesized receptors are retained in the endoplasmic reticulum (ER) and ultimately degraded [43] and a majority of the remainder are delivered to intracellular sites, with only a paucity going to the cell surface [44]. In their 2008 study, Decaillot and colleagues [17] show that a similar pattern of intracellular retention occurs with M/DOR and that its surface availability is dependent on chaperoning. The authors used HEK293 and Neuro2A expression systems to show that coexpression of MOR and DOR leads to reduced cell surface MOR. This reduction in surface MOR correlated with increased MOR and DOR colocalization with the Golgi apparatus, but not the ER. That is, coexpression of MOR and DOR led to the formation of M/DOR, which was retained in the Golgi. The authors then coexpressed MOR and DOR with RTP4, a member of the RTP family of chaperone proteins, which have been described to facilitate export of proteins, which accumulate in the Golgi [45]. Coexpression with RTP4 resulted in a redistribution of MOR and DOR, presumably as M/DOR, out of the Golgi to the cell surface. Notably, RTP4 had no effect on MOR when expressed alone, nor did RTP2 (another family member) have any effect. The authors then showed that RTP4 coimmunoprecipitates with MOR and DOR (but not c-terminal truncated MOR) and that RTP4 coexpression decreases MOR ubiquitination. Interestingly, while RTP4 coexpression normalizes (*i.e.*, increases) MOR surface abundance, the authors also showed it to decrease DAMGO (a MOR agonist) signaling, further suggesting that the increase in MOR surface abundance is in the form of M/DOR. (M/DOR functionality is discussed in detail in Section 4.) 

#### Summary, Protein Chaperone

**Figure 1:**
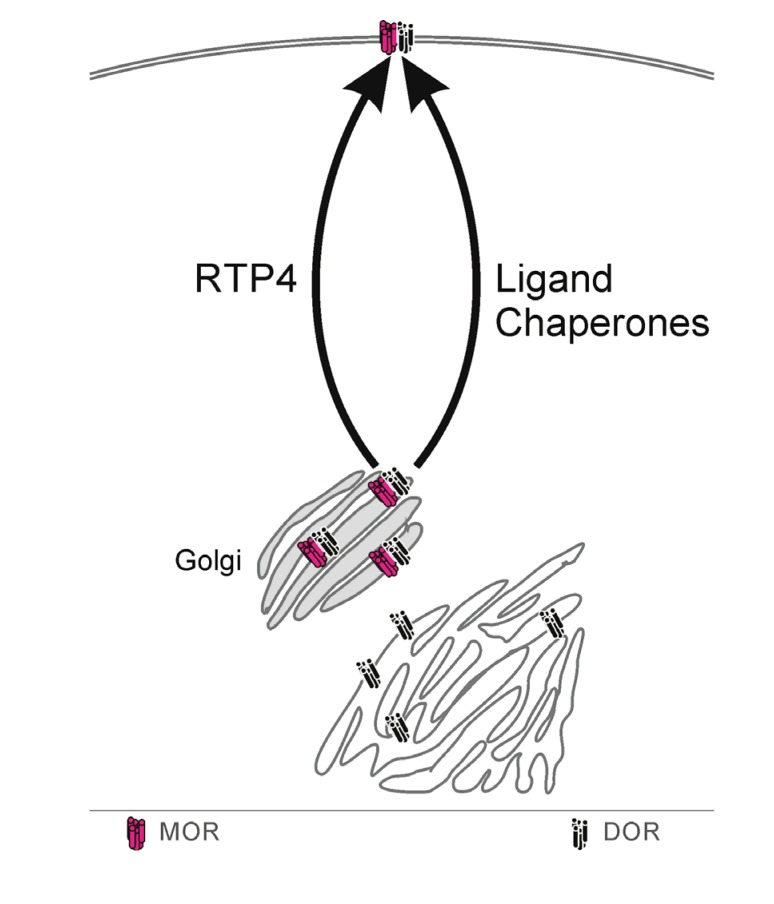
Outwards trafficking. Under basal conditions M/DOR is normally retained in the Golgi (grey) and surface availability is low. The augmentation of M/DOR surface availability involves transport chaperones, including RTP4 and, potentially, membrane-permeable ligands.

M/DOR can form intracellularly but is largely retained in the Golgi, ubiquitinated, and degraded. RTP4, a known chaperone protein, can interact with M/DOR to protect it from ubiquitination and chaperone its export from the Golgi to the cell surface. RTP4, and possibly other such chaperone proteins, thus, act to regulate M/DOR availability (Figure 1).

These findings, together with those showing increased M/DOR abundance following chronic morphine in the absence of changes in DOR mRNA, show that M/DOR availability, rather than formation, is the major regulated step controlling M/DOR abundance. That is, M/DOR forms readily, but is retained during post-ER transport (and ultimately degraded). This retention controls M/DOR availability and, thus, abundance.

### 3.2. Perspective from DOR Results

To date, the work by Decaillot and colleagues remains the only description of M/DOR chaperoning. However, it shares many conceptual and mechanistic similarities with reports of DOR chaperoning. Using metabolic pulse-chase labeling of DOR expressed in HEK293 cells, Petaja-Repo and colleagues [46] assessed DOR export from the ER compartment where it is typically retained. The authors identified several ligands, both agonists and antagonists, which acted as pharmacological chaperones of DOR. Of the compounds tested, all of the non-peptidic (*i.e.*, small molecule) ligands but none of the peptidic ligands acted as DOR chaperones. The authors conclude that the pharmacological chaperoning of DOR requires membrane permeability in order for the ligands to bind to immature DOR retained in the ER. It is speculated that ligand binding assists in proper folding of DOR, and thus its continued maturation and export.

Similarly, the same group went on to demonstrate [47] a role for palmitoylation, a post-translational lipid modification, of DOR in its ER export and trafficking to the cell surface. By preventing palmitoylation, the authors inhibited DOR transport to the cell surface. The authors also demonstrated, by inhibiting post-ER transport, that palmitoylation of immature DOR occur around the time of export from the ER. This leads to the conclusion that palmitoylation is a required step in the maturation of DOR and its successful release from the ER.

Despite the parallels, the potential role of DOR transport-regulating mechanism in M/DOR transport has not been investigated. In particular, the ability of non-peptidic ligands to act as DOR pharmacological chaperones has interesting implications for the role of chronic morphine treatment in increasing M/DOR abundance.

#### Summary, Perspective from DOR Results

The outwards trafficking of M/DOR appears to have many similarities to that of DOR, which is also retained intracellularly (albeit in the ER rather than the Golgi) and who’s outwards trafficking may be modulated by chaperones. Membrane-permeable ligands can act as DOR chaperones. DOR export is also modulated by palmitoylation. The roles of these systems in M/DOR export are unknown.

## 4. Function

As a result of controls on their availability, M/DOR appear to be present in consequential amounts only in certain states (e.g., prolonged opioid use). Interestingly, these states often present difficulties for classical opioid therapy (*i.e.*, reductions in efficacy). Considerable attention is being paid to M/DOR’s possible role in these changes and as its use as a novel therapeutic target. Research along both of these paths is elucidating the functional consequences of M/DOR existence, though interpretation of these results is made difficult by the variability of M/DOR availability.

### 4.1. Surface Expression

Current understandings of the effects of M/DOR availability on MOR and DOR surface expression are incomplete. As discussed earlier, Decaillot and colleagues [17] used a heterologous system to demonstrate that coexpression of DOR with MOR resulted in a reduction of surface MOR compared to cells expressing MOR alone. This change was attributed to the formation of M/DOR, with the resulting heteromers being retained in the Golgi. A substantial proportion of the MOR expressed was unavailable, which resulted in the reduction in MOR surface expression. These findings are contrasted by work by Walwyn and colleagues [48] using primary cultures of DRG neurons from wild-type and DOR knock-out (KO) animals. Measuring immunofluorescent labeling of MOR surface expression by flow cytometry, the authors show reduced MOR surface expression in DOR KO neurons. While *prima facie* contradictory, these results likely represent M/DOR effects on MOR surface expression in different conditions of M/DOR availability. In a simple expression system, MOR and DOR are produced at a near 1:1 ratio in large quantities, due to the use of CMV promoters. This appears to be ideal for M/DOR formation, with so much MOR diverted to M/DOR (and thus substantially retained in the Golgi, in part due to the lack of chaperones) that MOR surface expression is greatly reduced. In the more physiologically relevant DRG neuronal culture, MOR and DOR expression levels are far lower, M/DOR formation would likely be far less common, M/DOR chaperones would be available, and normal MOR regulatory mechanism are present. As a result, a small pool of M/DOR is on the surface in wildtypes and absent in DOR KOs. Surface MOR, meanwhile, remains normally and independently regulated in both states. Total surface MOR labeling (M/DOR + MOR) is then reduced in DOR KOs.

#### Summary, Surface Expression

The apparently contradictory findings regarding the effects of M/DOR formation on MOR surface expression can be explained by methodological differences. Under basal physiological conditions, the surface population of M/DOR is likely small, but sufficient that in DOR KO neurons, the apparent surface population of MOR is reduced by the loss of the M/DOR pool. The remaining MOR surface population remains under normal controls, present in physiologically relevant neurons but not in expressions system. This is further evidence that M/DOR availability is specifically regulated and not simply the result of stochastic MOR-DOR contacts. That is, M/DOR and MOR are independently regulated populations.

### 4.2. Ligand Binding

Much of the interest concerning the M/DOR heteromer stems from its unique pharmacology. Having both MOR and DOR binding sites and affinities for both MOR and DOR ligands, M/DOR pharmacology is, nevertheless, not simply a combination of the profiles of MOR and DOR. Fan and colleagues [15] showed that the ligand binding profile of M/DOR is distinct from those of MOR and DOR. The authors used competitive radioligand binding to assess the affinities of three OR agonists in cells expressing MOR alone, DOR alone, or MOR and DOR. DAMGO (a MOR agonist) and DPDPE (a DOR agonist) were shown to have high affinity in matched single OR-expressing cells, despite having far lower affinities than Deltorphin II (DELT, a DOR agonist) in MOR-DOR coexpressing cells. Thus, while binding the same overall class of ligands (opioids), M/DOR has a unique pharmacological profile. Prior to the discovery of widespread OR heteromerization, ORs were commonly classified into pharmacological subtypes. There was general consensus on the existence of eight such subtypes (MOR1-3, DOR1-2, KOR1-3), each with unique pharmacological profiles. The subtype classifications have fallen out of use since the identification of only a single cDNA (and, so, mRNA) for each OR and the failure to identify post-translational modifications, which might account for the various subtypes. It now appears that various OR heteromers may, in fact, be those pharmacologically distinct subtypes. Portoghese and Lunzer [49] identify the DOR1 subtype as a D/KOR heteromer by studying the *in vivo* actions of the KOR antagonist norbinaltorphimine (norBNI). norBNI was previously held to be nonspecific as it inhibited the actions of DAMGO and DPDPE. The authors show that norBNI’s antagonism of DAMGO is a result of norBNI blocking the actions of dynorphin-A, of which release is promoted by DAMGO. Blockade of dynorphin-A by dynorphin-A antiserum results in little added antagonistic effect of norBNI on DAMGO. However, this is not the case for the DOR1 agonist DPDPE. Even in the presence of dynorphin-A antiserum, norBNI retains its full antagonistic action. Conversely, norBNI has little effect on the DOR2 agonist DELT. The identity of DOR1 as D/KOR is reinforced by work from the same group using KO animals [50]. norBNI is able to antagonise DPDPE in wild-type but not KOR KO animals. The authors suggest that KO of KOR prevents the formation of D/KOR, and thus norBNI’s ability to antagonise DPDPE, while DPDPE itself retains efficacy at other DOR sites. In this case, DPDPE appears to be heteromer-active but not heteromer-specific.

#### Summary, Ligand Binding

M/DOR’s overall pharmacological profile is unique and responsiveness to any given ligand is different from that of MOR or DOR. Still, M/DOR’s pharmacology overlaps that of MOR and DOR. These findings are a useful example of the difficulties involved in understanding the pharmacology of M/DOR. Even in the most artificial heterologous systems, it coexists with both MOR and DOR. Therefore, much of the work investigating M/DOR ligands is only able to identify M/DOR-active ligands. The identification of heteromer-specific ligands is more demanding and frequently involves the synthesis and screening of novel compounds. For that reason, much attention has been paid to the use of ligand combinations, as well as bivalent ligands, to exploit heteromers (discussed below).

#### 4.2.1. M/DOR-Active Ligands

A large number of ligands to MOR or DOR have been reported to show activity at M/DOR and a summary is shown in Table 1. In some cases, these reports have also sought to reconcile previous categorizations of ligand by OR subtype activity with M/DOR. These studies are, however, limited by the difficulties in isolating M/DOR effects from MOR or DOR actions, as discussed previously.

**Table 1:** Ligands reported to be active at M/DOR.

Ligand	Monomer action	Reported heteromer action	Methods	Source
ADL5859	DOR agonist	M/DOR agonism (reduced potency)	G-protein activation screening assay	[51]
ARM1000390	DOR agonist	M/DOR agonism (reduced potency)	G-protein activation screening assay	[51]
Deltorphin II	DOR agonist	M/DOR agonism	Competitive binding	[15]
Deltorphin II	DOR agonist	M/DOR agonism	Calcium mobilization	[52]
Deltorphin II	DOR agonist	M/DOR agonism	G-protein activation screening assay	[51]
DPDPE	DOR agonist	none	Competitive binding	[15]
DPDPE	DOR agonist	M/DOR agonism	Calcium mobilization	[53]
DPDPE	DOR agonist	M/DOR agonism	Calcium mobilization	[52]
SNC80	DOR agonist	M/DOR agonism	Calcium mobilization	[54]
SNC80	DOR agonist	M/DOR agonism (reduced potency)	G-protein activation screening assay	[51]
DAMGO	MOR agonist	none	Competitive binding	[15]
DAMGO	MOR agonist	M/DOR agonism	Calcium mobilization	[53]
DAMGO	MOR agonist	M/DOR agonism	Calcium mobilization, GTPɣS binding	[52]
Methadone	MOR agonist	M/DOR agonism	Biotin protection, calcium mobilization	[55]
Morphine	MOR agonist	M/DOR agonism	Calcium mobilization, GTPɣS binding	[52]
Endomorphin-2	MOR agonist	M/DOR agonism	Inhibition of forskolin-evoked cAMP	[42]
Bremazocine	KOR agonist	M/DOR partial agonism	Calcium mobilization	[52]
U69593	KOR agonist	none	Calcium mobilization	[52]
Naltriben	DOR antagonist	Blocks M/DOR endocytosis but not signaling	Biotin protection, calcium mobilization	[55]
DAMGO & Deltorphin II	MOR agonist & DOR agonist	M/DOR agonism	GTPɣS binding	[56]
DAMGO & Deltorphin II	MOR agonist & DOR agonist	none	Antinociception	[57]
DAMGO & DPDPE	MOR agonist & DOR agonist	none	Antinociception	[57]
DAMGO & TIPPψ	MOR agonist & DOR antagonist	M/DOR agonism	GTPɣS binding	[56]
DAMGO & TICPψ	MOR agonist & DOR antagonist	M/DOR agonism	Antinociception	[57]
DAMGO & TIPP	MOR agonist & DOR antagonist	M/DOR agonism	Antinociception	[57]
DAMGO & Naltriben	MOR agonist & DOR antagonist	M/DOR agonism	GTPɣS binding	[56]
DAMGO & ICI 174,864	MOR agonist & DOR inverse agonist	M/DOR agonism	GTPɣS binding	[56]
Morphine & Deltorphin II	MOR agonist & DOR agonist	M/DOR agonism	GTPɣS binding	[56]
Morphine & TIPPψ	MOR agonist & DOR antagonist	M/DOR agonism	GTPɣS binding, cAMP inhibition, antinociception	[56]
Morphine & Naltriben	MOR agonist & DOR antagonist	M/DOR agonism	GTPɣS binding	[56]
Morphine & Naltrindole	MOR agonist & DOR antagonist	M/DOR antagonism	Calcium mobilization, antinociception	[58]
Morphine & ICI 174,864	MOR agonist & DOR inverse agonist	M/DOR agonism	GTPɣS binding	[56]
Fentanyl & Deltorphin II	MOR agonist & DOR agonist	M/DOR agonism	GTPɣS binding	[56]
Fentanyl & TIPPψ	MOR agonist & DOR antagonist	M/DOR agonism	GTPɣS binding	[56]
Fentanyl & Naltrindole	MOR agonist & DOR antagonist	M/DOR antagonism	Calcium mobilization, antinociception	[58]
Methadone & Deltorphin II	MOR agonist & DOR agonist	M/DOR agonism	GTPɣS binding	[56]
Methadone & TIPPψ	MOR agonist & DOR antagonist	M/DOR agonism	GTPɣS binding	[56]
Methadone & Naltrindole	MOR agonist & DOR antagonist	M/DOR antagonism	Calcium mobilization, antinociception	[58]
MDAN 16 to 21	Novel bivalent ligand	M/DOR agonism	Antinociception	[59]
CYM51010	Novel ligand	M/DOR agonism	M/DOR activation screening assay	[60]

#### 4.2.2. M/DOR Specific/Selective Ligands

The vast majority of the ligands reported to have activity at M/DOR are also active at either MOR or DOR. There is typically some difference in affinity or potency at the different receptor species. From this, some authors have concluded some of these ligands to be M/DOR selective [52], though most reports describe activities at M/DOR equal to or less than that at MOR or DOR. The identification of ligands, which are M/DOR specific, may not be possible given the nature of M/DOR. Even the identification of truly selective M/DOR ligands is difficult and limited by the challenge of interpreting findings derived from systems with both MOR and DOR present in addition to M/DOR. Two groups have now described high-throughput assays intended to screen compounds for M/DOR selectivity. (These screening assays are discussed in greater detail below.) Gomes and colleagues [60] report identifying such compounds with their assay. Briefly, the authors use four cell lines stably expressing MOR, DOR, MOR and DOR, or 5HT_5A_. In these cell lines, receptor activation results in the unmasking of beta-galactosidase activity, which then activates a chemiluminescent reporter. This provides the authors with cells, which report activity at MOR, DOR, MOR, and/or M/DOR, or 5HT_5A_. (In cells expressing MOR and DOR, the reporter is linked to MOR, meaning those cells report activity at both MOR and M/DOR.) The authors used these reporter cells to screen a small molecule library of several hundred thousand compounds for activity at M/DOR, but not MOR, DOR, or 5HT_5A_. From this library, 94 compounds were identified with an EC_50_ in M/DOR expressing cells of ≤10 µM and five times higher than the EC_50_ in MOR or DOR expressing cells. Of the 94 compounds meeting selectivity criteria in high-throughput screening, the author chose 14 for follow-up validation. Of these 14, six show higher efficacy in M/DOR expressing cells compared to MOR or DOR expressing cells. This hit rate likely reflects the high-throughput assay’s detection of compounds with higher M/DOR potency but not higher efficacy. The authors proceeded to characterise one of the identified compounds, CYM51010. They show a reduction in CYM51010 activity by coadministration of a M/DOR antibody (described above) but not an anti-Flag antibody. Indeed, coadministration of CYM51010 with both the M/DOR antibody and naltrexone (a MOR antagonist), completely blocks CYM51010 activity. *In vivo*, CYM51010 is antinociceptive and can be blocked by the M/DOR antibody. The susceptibility of CYM51010 to antagonism by the M/DOR antibody is an important finding in discriminating a ligand with activity at M/DOR from one active at only MOR or DOR. This technique addresses one of the major limitations of other studies seeking to identify M/DOR-active ligands in which changes in activity in M/DOR expressing cells could not distinguish between those changes being a result of M/DOR activity or a result of changes in MOR or DOR availability or coupling.

#### Summary, M/DOR Specific/Selective Ligands

Most of the M/DOR active ligands identified to date are also MOR or DOR ligands, which is to be expected because of the similar/shared binding sites and the availability of MOR and DOR ligands. While the activities of these ligands at M/DOR differ from the activities of the same ligands at either MOR or DOR, they generally cannot be said to be M/DOR selective or specific. The identification of true M/DOR selective/specific ligands depends on recently-begun projects which have implemented novel screening assays. A preliminary report from one group has identified one novel M/DOR selective agonist, CYM51010.

#### 4.2.3. Ligand Combinations

One of the more conceptually straightforward approaches to exploiting a pharmacological target with two binding sites has been to simply use two ligands. In their 2004 work, Gomes and colleagues [56] show in cell lines coexpressing MOR and DOR, that coadministration of DELT with morphine or DAMGO produces increased GTPɣS binding *versus* administration of morphine or DAMGO alone. This, of course, could be explained by additive effects at MOR and DOR separately. However, the authors proceed to demonstrate similar increases in signaling follow MOR agonist coadministration with TIPPψ (peptidic DOR antagonist), naltriben (non-peptidic DOR antagonist), and ICI 174,864 (DOR inverse agonist). They go on to show the same effects in mouse spinal cord membranes and then demonstrate the augmentation of morphine antinociception, *in vivo*, following TIPPψ coadministration. These findings lead to the conclusion that simple occupancy of DOR binding sites by any ligand is sufficient to augment MOR signaling. 

These findings are reinforced by the work of Szentirmay and colleagues [57], who investigated the *in vivo* coadministration of DAMGO with DOR ligands. Interestingly, they conducted these experiments in both naive and chronic morphine treated animals. Chronic morphine treatment causes both morphine tolerance (and cross-tolerance to other MOR agonists) and the increased availability of M/DOR (discussed above). In morphine treated mice, the potency of DAMGO antinociception is, unsurprisingly, greatly reduced (that is, there is tolerance). Coadministration of DELT (DOR agonist) had no effect on DAMGO potency, while DPDPE (DOR agonist) coadministration actually reduced DAMGO potency even further. Conversely, coadministration of either TIPP or TICPψ (both DOR antagonists) increase DAMGO potency, restoring it to control (non-morphine-tolerant) levels.

The mechanisms responsible for this phenomenon were addressed by the work of Rozenfeld and Devi [61], in which they describe the unique downstream coupling of M/DOR (discussed in detail below). Briefly, the authors show that M/DOR constitutively recruits β-arrestin2 and that subsequent DAMGO-evoked signaling results in an altered pattern of ERK phosphorylation. Coadministration of DAMGO with TIPPψ is shown to revert ERK phosphorylation to the pattern observed with MOR alone. There is, though, no evidence that ligand combinations dissociate M/DOR. Rather, MOR/DOR ligand combinations appear to shift M/DOR from β-arrestin2-mediated signaling to non-β-arrestin2-mediated signaling. This could be interpreted as a normalization of signaling from M/DOR-typical to MOR-typical. This would also explain the augmentation of MOR activity. In the specific case of the restoration of MOR activity following chronic morphine, an appealing explanation is that chronic morphine causes an increase in M/DOR availability. The shift in surface receptor complement from MOR to M/DOR results in decreased overall MOR responsiveness. This change contributes to observed MOR tolerance. Subsequent administration of MOR agonist - DOR antagonist combination can normalise M/DOR signaling. This presents as a reversal of tolerance.

This narrative is contrasted by a study which reports blockade of MOR signaling by DOR antagonist [58]. This work used HEK293 cells expressing either MOR alone or MOR and DOR. In MOR expressing cells, DOR antagonist naltrindole (NTI) is unable to induce calcium mobilization evoked by morphine, fentanyl, or methadone (all MOR agonists). In cells coexpressing MOR and DOR, however, NTI was able to block the effects of all three MOR agonists. *In vivo* assessments of antinociception produced by these MOR agonists also demonstrated the ability of NTI to shift dose-response curves rightward in a dose-dependent manner. That is, increasing doses of NTI resulted in increasing reductions in MOR agonist antinociception. These results are difficult to reconcile with other reports of ligand combinations acting on M/DOR. The most apparent difference in the models used is that these findings are from opioid-naive cells and animals, where as other reports showing augmentation of MOR activity by DOR antagonist show those effects in opioid tolerant states. It is also possible that these results reflect pharmacological chaperoning of DOR by the non-peptidic NTI which did not occur with peptidic TIPP, TIPPψ, or TICPψ.

#### Summary, Ligand Combinations

The majority of current work aiming to rationally manipulate M/DOR has used combinations of MOR and DOR ligands. Activity by MOR agonists at M/DOR is less than that at MOR. Coadministration of a MOR agonist with a DOR ligand can result in augmented MOR activity at M/DOR. Peptidic DOR antagonists appear to be the most effective.

Shifts in surface receptor complement towards more M/DOR and less MOR may contribute to MOR agonist tolerance. High efficacy activation of M/DOR by appropriate ligand combinations can manifest as a reduction of tolerance (*versus* low efficacy activation by single MOR ligands).

#### 4.2.4. Bivalent Ligands

A particularly interesting method to exploit M/DOR via ligand combinations has been the design of so-called bivalent ligands. These compounds are constructed of two distinct pharmacophores joined by a spacer into a single molecule. Essentially, these are single molecule ligand combinations. The use of bivalent ligands targeted against OR heteromers was first described, and subsequently developed, for D/KOR [62,63]. This technique was translated to M/DOR by Daniels and colleagues [59], who designed their strategy based upon the reports of the M/DOR efficacy of MOR agonist-DOR antagonist combinations (see Section 4.2.3 above). The resulting bivalent ligands were named MDANs (MOR-agonist-DOR-antagonist) and used the oxymorphone (MOR agonist) and naltrindole (DOR antagonist) pharmacophores. Based on previous experience with D/KOR and M/DOR structural models, the authors expected that MOR-DOR heteromerization should result in MOR and DOR binding pockets fixed in space relative to each other. That is, the binding pockets should remain a set distance away from each other, as dictated by the structure of the heteromer. This distance necessitates the spacer in MDANs, linking the two pharmacophores and holding them at a set distance away from each other. As the authors did not know the precise spacing of the binding pockets, they constructed a series of MDANs with spacers varying from 16 to 21 atoms in length. The authors then tested the *in vivo* antinociceptive efficacy of these ligands. Administered acutely, none of the MDANs is as potent as a control ligand containing only a MOR-agonist pharmacophore (a ‘monovalent’ ligand) or the parent compound, oxymorphone. This finding is consistent with previous reports of ligand combinations in naive animals (a state which appears to have low M/DOR availability). However, when administered chronically, the bivalent ligands cause less tolerance and physical dependence than the monovalent control, the monovalent control combined with a separate DOR antagonist, or morphine. Interestingly, MDANs with the longest spacers cause no detectable tolerance and nor significant physical dependence. 

In a follow-on study, the rewarding properties of the longest MDANs were investigated [64]. These MDANs have spacers of 19 and 21 atoms (MDAN-19 and MDAN-21, respectively). MDAN-16 was included as a control. As reported, conditioned place preference, a measure of drug reward, develops to morphine, fentanyl (a MOR agonist), and the monovalent control, but not to MDAN-19 or MDAN-21. Results for MDAN-16 were equivocal and all ligands were administered at a dose four-fold higher than their ED90 dose for antinociception. The place preference induced by the monovalent control could be blocked by coadministration of naloxone (a MOR antagonist) but not by coadministration of a DOR antagonist. This demonstrates that inability of MDAN-19 and -21 to induce a place preference is not simply a matter of ligand combination, but rather that it is dependent on the bivalent nature of the ligands. The authors also demonstrate that MDAN-19 and -21 are similarly unable to reinstate a previously extinguished place preference to morphine, whereas reinstatement does occur with both morphine and the monovalent control. Together, these results show that MDAN-19 and -21 are less rewarding than typical MOR agonists. These measures of reward are widely used as animal models of abuse liability. Overall, long-spacer MDANs are suggested to be effective analgesics with reduced tolerance, physical dependence, and psychological dependence.

#### Summary, Bivalent Ligands

The construction of bivalent ligands is a refinement of the ligand combination concept. Bivalent ligands allow more specific targeting of heteromers. Optimizations of pharmacophore spacing have yielded insights into the relative orientation of heteromer binding pockets. The M/DOR bivalent ligands studied to date have involved a single MOR agonist pharmacophore (oxymorphone) linked to a single DOR antagonist pharmacophore (naltrindole; these compounds are termed MDAN for Mu-Delta-Agonist-aNtagonist). One can imagine that future studies using the bivalent ligand concept could investigate different specific pharmacophore combinations.

MDANs have less acute efficacy than monovalent MOR agonists, both in terms of antinociception and reward. Administered chronically, they cause less tolerance and physical dependence.

### 4.3. Downstream Coupling

The intracellular signaling cascades to which M/DOR couples are poorly understood. Broadly, M/DOR acts similarly to its constituent ORs: M/DOR activation causes activation of G-proteins, inhibition of intracellular calcium release, and overall inhibition of cellular excitability. In the majority of work addressing M/DOR, these effects are used as accessible telltales for the overall concept of receptor functionality. The lack of attention to M/DOR downstream coupling is both understandable, given the often intimidating complexities of intracellular signal propagation, and lamentable, because it is now abundantly clear that M/DOR is a distinct functional species that behaves differently, often very much so, than other OR species. The differences in the physiological actions of M/DOR certainly involve changes in downstream coupling. Research which has addressed M/DOR downstream coupling remains limited in both quantity and scope.

Fan and colleagues [15] sought to identify the G-protein to which M/DOR couples. They used a heterologous system to generate cells expressing MOR, DOR, or MOR and DOR (and so, presumably, M/DOR). In cells expressing MOR and DOR, pertussis toxin failed to abolish the inhibition of adenylyl cyclase activity caused by DELT (a DOR agonist). This is in contrast to cells expressing only MOR or DOR, in which pertussis toxin did abolish OR functionality. MOR and DOR are commonly held to signal via coupling with the pertussis toxin-sensitive Gα_i_. These data suggest that M/DOR signals, at least in part, via coupling with a pertussis toxin-insensitive G-protein. The authors then radiolabeled activated G-proteins with GTPɣ^35^S and immunoprecipitated Gα_i3_ and G_z_ (a pertussis-insensitive G-protein capable of inhibiting adenylyl cyclase). In doing so, they showed that in cells expressing only MOR or DOR, only Gα_i3_ was activated. In cells expressing MOR and DOR, both Gα_i3_ and G_z_ were activated. Therefore, it appears that MOR and DOR couple only to Gα_i3_, whereas M/DOR couples, in part or in whole, with G_z_. 

As discussed above, Rozenfeld and Devi [61] investigated M/DOR association with β-arrestin2. These authors also used a heterologous system to generate cells expressing MOR, DOR, or MOR and DOR (and so, presumably, M/DOR). In cells expressing MOR and DOR, β-arrestin2 appears to colocalize with MOR and DOR. This contrasts with what appears to be nearly no colocalization in cells expressing only MOR or DOR. More convincingly, β-arrestin coimmunoprecipitates with MOR and DOR in coexpressing cells, but not with either MOR or DOR when expressed alone. The authors note that the association of β-arrestin2 with MOR and DOR even in the absence of ligand binding or activation suggests that M/DOR constitutively recruits β-arrestin2. Given that β-arrestin2 has previously been shown to be involved an the ERK signaling pathway, the authors then assessed the time course of ERK phosphorylation following MOR agonist treatment in cells expressing either MOR and DOR or MOR alone. They showed that ERK phosphorylation occurs at later time points in cells expressing MOR and DOR, compared to cells expressing MOR alone. That is, the time course of ERK phorphorylation is right-shifted. Interestingly, this shift in ERK phorphorylation is proportionate to the ratio of MOR to DOR expressed; relatively more MOR results in less of a shift (*i.e.*, closer to pattern of phorphorylation seen with MOR alone), while shifting MOR expression closer to a 1:1 ratio with DOR results in a progressively greater right-shift in ERK phosphorylation. This suggests that M/DOR activation results in a predominately late-phase ERK phorphorylation, in contrast to the predominately early-phase phosphorylation seen following MOR activation. Using the PKC inhibitor calphostin C, the authors then showed that the early phase of ERK phosphorylation seen with MOR is PKC-dependent; early ERK phosphorylation is inhibited by calphostin C. Late phase ERK phosphorylation as seen with M/DOR, however, is not PKC-dependent. Though not PKC-dependent, the shift to late-phase ERK phosphorylation does appear to be β-arrestin2-dependent. Knock-down of β-arrestin2 expression by siRNA in cells expressing MOR and DOR results in restoration of ERK phorphorylation to the pattern observed in cells expressing only MOR. As described above, the authors also demonstrated that this β-arrestin2-mediated signaling occurs in response to single-ligand M/DOR activation but is abolished in favor of MOR-typical signaling in response to some ligand combinations. 

These studies both offer interesting glimpses of M/DOR downstream coupling and its differences from that of MOR and DOR. In particular, the work of Rozenfeld and Devi [61] highlights that not only does M/DOR have distinct coupling, but that that coupling can change in an agonist-dependent manner. Indeed, the emerging concept of unique agonist-dependent effects now appears to apply to ORs generally. That two drugs of the same class may act via the same receptor and have different downstream effects - sometimes slightly but sometimes substantially – is challenging to understand but presents promising avenues for therapeutic exploitation. However, these studies specifically and the current understandings of M/DOR intracellular signaling more generally remain narrow in scope. The identification of associations with a handful of intracellular signaling components and changes in a few downstream effects are interesting and important progress but remain well short of understanding the full cascades and variations involved.

#### Summary, Downstream Coupling

**Figure 2:**
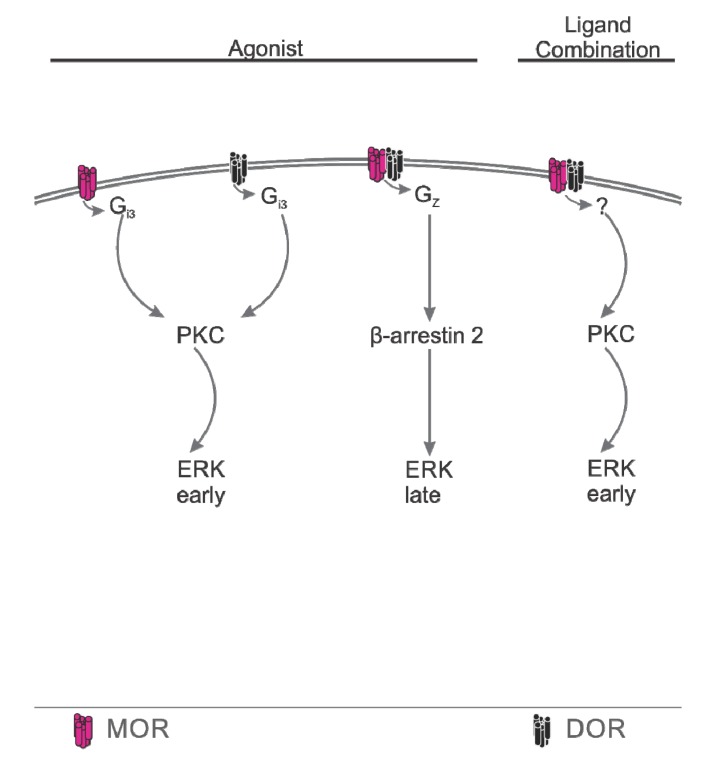
Downstream coupling. MOR and DOR monomers, when activated by their respective ligands, signal via activation of Gi3, which leads, via PKC, to early phase ERK phosphorylation. M/DOR ligand-induced signaling occurs via G_z_ and β-arrestin2 and involves a shift to late phase ERK phosphorylation. Activation of M/DOR by ligand combinations (MOR agonism, DOR antagonism) results in monomer-like downstream coupling resulting in PKC-dependent early phase ERK phosphorylation

M/DOR has distinct downstream coupling that can involve signaling via G_z_ and/or β-arrestin2. Subsequent ERK phosphorylation is temporally shifted in M/DOR signaling *versus* that of MOR or DOR. Such distinct coupling can change in an agonist-dependent manner. Activation by certain ligand combinations appears to shift M/DOR downstream coupling to that of MOR or DOR (Figure 2). These findings correspond with the previously described cell- and system-level effects of ligand combinations/bivalent ligands.

## 5. Inwards Trafficking

In addition to availability and functionality at the surface, OR inwards trafficking is fundamental to the function of ORs. OR desensitization, internalization, and recycling to the plasma membrane have significant functional consequences, affecting both analgesia and tolerance [65,66,67,68] (for reviews [69,70,71,72]). ORs routinely internalise upon agonist exposure. Such internalization is likely agonist-directed and, together with post-internalization handling, modulates cell-level OR functionality. Following endocytosis, receptors may undergo recycling back to the plasma membrane, which is thought to underlie re-sensitization, or may undergo targeting to the degradation pathways, a key process in receptor down-regulation. As these fates are mutually exclusive, GPCRs are generally categorized as either ‘class A’ (recycled) or ‘class B’ (degraded) [73]. This division appears to arise from the relative stability of the GPCR-arrestin interaction. Individual receptors are direct to one or the other pathway based upon ubiquitination signals, with greater ubiquitination marking the receptor for degradation. The post-internalization handling of M/DOR remains unresolved, however, as its constituents are trafficked differently. While both MOR and DOR are internalized constitutively and in response to activation by agonists, MOR is recycled to the cell surface (class A), whereas DOR is trafficked to lysosomes (class B) and degraded [70,74,75].

Two recent studies have begun to elucidate the post-internalization trafficking of M/DOR. He and colleagues [27] report that MOR and DOR cointernalize in response to agonist exposure. Using a HEK293 expression system, the authors immunolabeled surface MOR and DOR (and so, presumably, M/DOR). Following deltorphin I, deltorphin II, SNC80 (all DOR agonists), or DAMGO (a MOR agonist), MOR and DOR were both observed to internalise more than in control conditions and appeared to be located in similar areas within the cell. The authors went on to use a LysoTracker probe to additionally label acidic organelles and report that following treatment with deltorphin I, but not DAMGO, MOR and DOR appeared to colocalize with these lysosome-like acidic organelles. Immunoblotting was then used to show that deltorphin I, but not DAMGO, increased ubiquitination of coimmunoprecipitated MOR and DOR (a signal for trafficking to degradation).

Similarly, Milan-Lobo and Whistler [55] also used a HEK293 expression system and report that in cells coexpressing MOR and DOR, both methadone and DAMGO (both MOR agonists) induced the endocytosis of DOR. This effect was not observed in cells expressing only DOR. The authors used serial immunoprecipitations to identify the internalized species and report that both methadone and DAMGO induced the endocytosis of MOR and M/DOR, but not DOR. The authors then showed that this methadone- and DAMGO-induced endocytosis was blocked by pretreatment of the cells with naltriben (a DOR antagonist). Interestingly, although naltriben was able to block methadone- and DAMGO-induced endocytosis, it was only moderately effective at inhibiting methadone and DAMGO signaling. Indeed, naltriben was more effective at inhibiting methadone and DAMGO signaling in cells expressing only MOR than in the cells coexpressing MOR and DOR. This is interesting, as it is further evidence that MOR-agonist/DOR-antagonist ligand combinations act to stabilize M/DOR while still permitting signaling. Precise interpretations are difficult, however, as it is unclear whether naltriben’s effects on MOR agonist signaling owed to effects at MOR or M/DOR. Additionally, these findings also raise the question of naltriben’s selectivity at the concentrations used.

**Figure 3:**
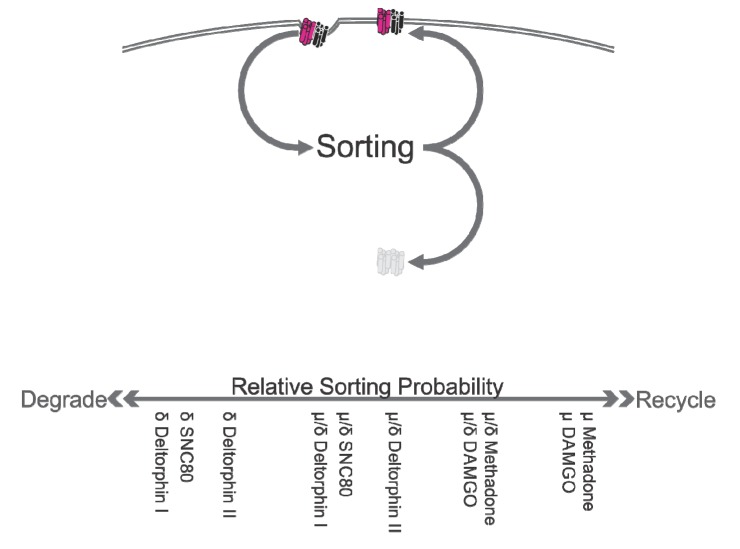
MOR, DOR, and M/DOR are internalized in response to agonist activation. The sorting of receptors to either recycling or degradation is probabilistic and varies by both receptor species and ligand. Monomeric MOR and DOR have high probabilities of recycling and degrading, respectively. M/DOR is intermediate to the monomers and is subject to greater ligand biases.

Puzzlingly, the findings of Milan-Lobo and Whistler [55] do not fully agree with those of He and colleagues’ [27] with regards to the post-internalization fate of M/DOR. Milan-Lobo and Whistler used biotinylation to assess the degradation of internalized receptors and serial immunoprecipitation to identify the species of those degraded receptors. In response to methadone treatment, both MOR and M/DOR were endocytosed. Internalized MOR were relatively stable, with only ~25% degraded after two hours’ agonist treatment. Internalized M/DOR, however, underwent significantly greater degradation, with ~85% degraded after two hours. This contrasts with He and colleagues’ report that treatment with DAMGO did not result in either increased ubiquitination or lysosome-like acidic organelle colocalization or MOR and DOR. This discrepancy may owe to the different ligands used by the two groups, methadone and DAMGO (summarized in Table 2). If this were the case, it would suggest that M/DOR undergoes agonist-directed post-internalization trafficking. It is also possible that this inconsistency reflects a methodological limitation of He and colleagues’ study, which relied on a coarse and somewhat subjective measure of OR-lysosome associations. Finally, it is conceivable that M/DOR post-internalization trafficking does not cleanly fit the model of either MOR or DOR but is, rather, intermediate, with trafficking to degradation occurring probabilistically, not obligately (Figure 3). In such a case, the alternative fate is recycling. In a state of continued agonist exposure, recycled receptors would soon repeat the same probabilistic sorting. Therefore, the original pool of receptors would inevitably, albeit gradually, be fully depleted by trafficking to degradation. Thus, post-internalization trafficking intermediate to those of MOR and DOR would appear, ultimately, to be ‘class B’ trafficking to degradation but would occur at a slower rate. If this were the case, the discrepancy between these findings might simply be a matter of time point, with early assessments concluding little degradation and later assessments finding near-complete degradation. Further, the amount of M/DOR associated with lysosomal compartments at any given time would be less than expected for class B receptors. Studies using measures of lysosomal colocalization would be biased towards concluding a lack of trafficking to degradation. This illustrates the need for further and more detailed investigation of M/DOR post-internalization trafficking. In particular, both studies to address this topic have relied upon the use of HEK293 expression systems, which, while convenient, are limited by a lack of physiological relevance. 

**Table 2:** Ligands reported to be active at traffick M/DOR.

Ligand	Internalization	Degradation	Recycling	Source
Deltorphin I	Increased	Increased	-	[27]
Deltorphin II	Increased	-	-	[27]
SNC80	Increased	-	-	[27]
DAMGO	Increased	No change	-	[27]
DAMGO	Increased	-	-	[55]
DAMGO & Naltriben	No change	-	-	[55]
Methadone	Increased	Increased	-	[55]
Methadone & Naltriben	No change	-	-	[55]

“-” denotes untested conditions.

### Summary, Inwards Trafficking

MOR and DOR undergo different post-internalization trafficking: MOR are ultimately recycled and DOR are ultimately degraded. The handling of M/DOR is, therefore, difficult to predict because of the differential behavior of its constituents. Recent studies using different methodologies have reported inconsistent findings regarding M/DOR post-internalization trafficking. Some ligands induce M/DOR internalization. Post-internalization, M/DOR appear more likely to be degraded than MOR. The literature regarding M/DOR inwards trafficking remains limited.

## 6. Conclusions and Outlooks

M/DOR, and OR heteromers more generally, are extant and physiologically relevant receptor species. A plethora of evidence demonstrates them to participate in the cellular environment as would be expected of any distinct receptor type. They are not, obviously, a *de novo* novel receptor. However, conceptualizing M/DOR functionality in terms of a distinct receptor provides a useful framework with which to understand current findings. The functional availability of M/DOR is complexly regulated and responsive to cellular conditions. When present at the cell surface, M/DOR exhibits unique pharmacology; both ligand binding and responsiveness and downstream coupling are clearly distinct from other ORs, including the constituents. 

Notably, M/DOR appears to have a range of actions that vary in a ligand- (or ligands-) dependent manner. As such, current exploitations of M/DOR often involve combinations of ligands or pharmacophores.

The recent identification of OR crystallographic structures and the continued development of M/DOR specific tools hold considerable promise for the further understanding of M/DOR and its exploitation as a viable therapeutic target. Definitive OR crystallographic structures will permit true modeling of heteromer-forming OR-OR interactions, as well as providing a vastly improved understanding of ligand binding to all species of OR. This is of particular importance to those interested in the therapeutic potential of M/DOR, as such understandings will improve the rational design of new ligands, both for M/DOR and ORs more broadly (see [24]). Further driving the therapeutic targeting of M/DOR is the recent development of assays to screen for M/DOR active and selective compounds [51,60]. Both assays were designed to allow for high-throughput screening and together demonstrate the interest in designing better M/DOR ligands.

Concurrently, fundamental understandings of M/DOR are being advanced by the availability of new, M/DOR-specific tools. The generation of specific M/DOR monoclonal antibodies is facilitating studies with physiologically relevant and *in vivo* models [37]. This is an important step in moving M/DOR work beyond the constraints of heterologous expression systems and is part of uncovering the physiological role of M/DOR. Additionally, the development of polypeptides to competitively dissociate M/DOR provides an experimental functionality akin to knock-out or knock-down of classical receptor species [27]. This is an ability, which has not been available to M/DOR research because of the nature of heteromer; that is, one cannot knock-out M/DOR without also knocking-out MOR or DOR and thoroughly confounding any results.

With the development and availability of better tools for addressing M/DOR, including M/DOR-selective antibodies and ligands, the use of more appropriate model systems, including primary neuronal cultures, *ex vivo* tissues, and whole animals, is becoming increasingly feasible. These advancements are helping to drive the shift from addressing M/DOR as a solitary receptor to working identify its roles in the wider cellular and system contexts. This shift is required for, and will ultimately determine, the usefulness of M/DOR as a therapeutic target.
